# A simple and efficient purification method of native immunoreactive antigen for diagnosis of camel hydatidosis

**DOI:** 10.14202/vetworld.2020.141-146

**Published:** 2020-01-23

**Authors:** Nagwa I. Toaleb, Mohamed S. Helmy, Eman E. El Shanawany, Eman H. Abdel-Rahman

**Affiliations:** 1 Department of Parasitology and Animal Diseases, National Research Centre, El Buhouth St., Dokki, Cairo, Egypt; 2 Department of Molecular Biology, National Research Centre, El Buhouth St., Dokki, Cairo, Egypt

**Keywords:** camel hydatidosis, cystic echinococcosis, *Echinococcus granulosus*, gel filtration chromatography, hydatid cyst fluid, indirect enzyme-linked immunosorbent assay, sodium dodecyl sulfate–polyacrylamide gel electrophoresis, Western blot

## Abstract

**Background:**

Cystic echinococcosis (CE), a zoonotic disease that affects animal and human health, is of increasing economic importance due to high morbidity rates and high economic losses in the livestock industry.

**Aim:**

The present study was conducted to purify the antigen from hydatid cyst fluid (HCF) with high diagnostic efficacy of camel hydatidosis using indirect enzyme-linked immunosorbent assay (ELISA).

**Materials and Methods:**

The HCF antigen was purified using Sephacryl S-300 column chromatography. Characterization of fractions was performed using reducing and non-reducing sodium dodecyl sulfate–polyacrylamide gel electrophoresis (SDS-PAGE) and Western blot analysis. Further, antibodies against *Echinococcus granulosus* cysts in camel serum were detected using indirect ELISA.

**Results:**

The purification process resulted in three fractions of antigens: FI, FII, and FIII. Indirect ELISA showed that higher diagnostic efficacy was observed in FI than in FII and FIII. Indirect ELISA, in which FI was utilized, showed 88% sensitivity and 91.7% specificity. Non-reducing SDS-PAGE showed that FI had two bands of molecular weights 120 and 60 kDa. Western blot analysis of FI demonstrated that 60, 38, and 22 kDa were antigenic bands when reacted with naturally infected camel sera with *E. granulosus* cysts. Using indirect ELISA, F1 recorded an infection percentage of 81.7% in randomly collected camel serum samples.

**Conclusion:**

FI is a promising antigen for accurate diagnosis of camel CE using indirect ELISA.

## Introduction

Cystic echinococcosis (CE), a zoonotic disease caused by the larval stage of the *Echinococcus granulosus*, is an animal and human health problem of increasing economic and public health importance [1]. CE in animals is asymptomatic and its diagnosis is made at necropsy [2,3], which depends on the analysis of morphological features using light or electron microscopy [4,5]. The serological diagnosis of CE improves the quality of the management and treatment of the disease. Enzyme-linked immunosorbent assay (ELISA) has been useful for the diagnosis of CE in domestic animals and humans [6]. It is generally cheap, quick, and requires fewer trained and specialized personnel [7-9].

Commercially available immunodiagnostic kits often depend on crude antigen preparations of *E*. *granulosus* hydatid cyst fluid (HCF); hence, it lacks satisfactory specificity and sensitivity [10]. Unsatisfactory performances may be due to the poor quality of antigen preparations. To avoid this problem, novel tests using purified antigens have been utilized in previous studies [11,12]. Purification of HCF antigens is essential to remove cross-reactivity and increase the sensitivity of techniques for the detection of low levels of antibodies [13]. El Deeb *et al*. [14] reported that the detected antibody of *E*. *granulosus* HCF in sheep using different antigens showed that purified HCF antigen was the most effective antigen compared with excretory/secretory and somatic antigens of protoscolex. The response of HCF antigens depends on the host and the location of the parasitic cysts [10]. Furthermore, the specificity and diagnostic efficacy of purified HCF antigen were higher than those of protoscolex antigen in serological studies on CE among camels in Egypt [15].

Antigen B and antigen 5, the most immunogenic antigen among HCF antigens, play an important role in the life cycle of the cestode [16]. However, interestingly, antigen 5 is immunoreactive in all stages of CE pathology compared with antigen B, which reveals a reduced antibody capturing activity in all CE stages [17]. Moreover, antigen 5 is one of the most immunogenic proteins present in HCF. Pagnozzi *et al*. [18] obtained an HCF fraction highly enriched in native antigen 5 through fast protein liquid chromatography on a Superdex-200 column with strong reactivity in both ELISA and Western blotting assays. The enriched antigen 5 fraction obtained from concentrated aliquots of sheep HCF was used for the accurate diagnosis of human CE using ELISA [19].

This study aimed to adopt a simple and inexpensive HCF purification method to obtain purified antigen of high diagnostic potency for camel CE.

## Materials and Methods

### Ethical approval

All experimental procedures were performed according to the institutional guidelines of the National Research Centre’s Animal Research Committee under protocol number 18/198.

### Antigen preparation

The HCF antigen was prepared from hydatid cysts removed from the lungs of slaughtered camels in an abattoir in Cairo in accordance with Swarna and Parija [20]. Briefly, HCF was aseptically aspirated in a sterile tube. HCF was further centrifuged at 6000×g for 45 min at 4°C and then dialyzed against phosphate buffer saline with pH 7.2. Finally, HCF was centrifuged at 6000×g for 30 min. The supernatant was collected and its protein content was estimated using the Lowry *et al*. method [21].

### Preparation of hyperimmune rabbit serum

Antibodies against *E. granulosus* HCF antigen were produced in two healthy male New Zealand rabbits free of parasitic infections, weighing 1.5 kg, and about 2 months of age. Two rabbits were subcutaneously immunized with 40 μg/kg of crude HCF antigen emulsified in Freund’s complete adjuvant according to Guobadia and Fagbemi [22]. On day 14, another dose of antigen was injected in Freund’s incomplete adjuvant according to Fagbemi *et al*. [23]. The second and third booster doses were administered on days 21 and 28, respectively. After 4 days, the last blood samples were collected from the ear vein and serum samples were separated.

### Collection of serum samples

In total, 25 positive camel serum samples were collected from infected slaughtered camels with lung cysts during several visits to a local abattoir. Further, 12 blood samples were collected as negative samples from healthy camels free of cysts as confirmed by veterinary inspection. ELISA was used to diagnose 93 camel serum samples randomly collected from a local abattoir in Cairo. Sera were separated from all blood samples and kept at −20°C until use.

### Purification of HCF antigen by gel filtration

The concentrated solution containing the HCF antigen was applied onto a Sephacryl S-300 gel filtration column chromatography (142 cm in length and 1.75 cm in diameter). The column was equilibrated and fractions were eluted with 0.02 mol/l phosphate buffer saline (pH 7.2) containing 0.03 mol/l phenylmethylsulfonyl fluoride and 0.02% NaN_3_ at a flow rate of 30 ml/h. The eluted fractions were collected in 175 tubes of 2 ml each. Furthermore, the proteins were monitored by measuring the absorbance at 280 nm using a spectrophotometer (Shimadzu).

### ELISA

Diagnostic value of crude antigen and isolated fractions was compared using indirect ELISA with hyperimmune rabbit sera and naturally infected camel sera according to Santiago and Hillyer [24]. The most potent fraction was adopted to diagnose *E. granulosus* cysts in randomly collected camel sera. Checkerboard titration was used to determine the antigen concentrations and dilution of sera as well as protein A horseradish peroxidase (Sigma Chem. Co., St. Louis, USA). The cutoff values were measured as mean values +3SD [25].

### Characterization of fractions

Non-reducing sodium dodecyl sulfate–polyacrylamide gel electrophoresis (SDS-PAGE) was performed using 12% polyacrylamide gel [26] stained with silver stain [27], photographed, and analyzed using Molecular Imager Gel Doc™ XR+ with Image Lab Software (Bio-Rad, California, USA). Molecular weights of bands observed were calculated using molecular weight of standard proteins which were electrophoresed on the same gel.

### Immunoblot

After another electrophoresis, under reducing condition in 10% SDS-PAGE, eluted fractions, crude HCF antigens, and Prestained Protein Ladder (Vivantis Technologies) were blotted onto nitrocellulose membrane as described by Towbin *et al*. [28] in a blotting system. After washing and blocking, the membrane was incubated with diluted naturally infected camel sera (1:200). Protein A horseradish peroxidase conjugate was used at a dilution of 1:2000. In addition, bands were observed by adding 4-chloro-1-naphthol (Sigma), and the membrane was photographed by Molecular Imager Gel Doc™ XR+ with Image Lab Software.

### Statistical analysis

Sensitivity and specificity were calculated according to Gonzalez-Sapienza *et al*. [29], and the diagnostic parameters used were as follows: True-positive values (Tp), sera from camel naturally infected with *E. granulosus* as confirmed by parasitological examination; false-negative values (Fn), sera from camel infected with CE showing negative readings; false-positive values (Fp), sera from non-infected camels showing a positive result; and true-negative values (Tn), sera from healthy camels free of cysts as confirmed by veterinary inspection showing negative readings.

## Results

### Isolated fractions

**Figure-1:**
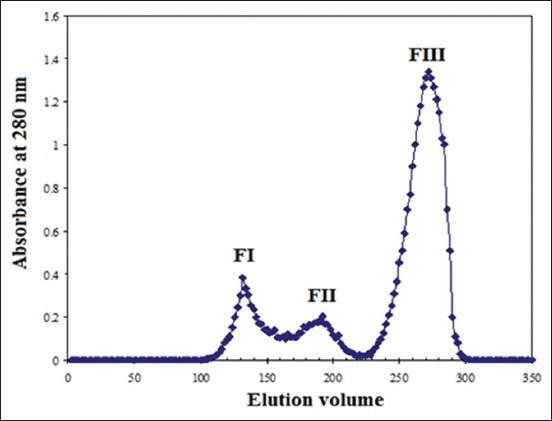
Purification profile of hydatid cyst fluid antigen on Sephacryl S 300 column chromatography.

The purification process resulted in the isolation of three fractions of antigens: FI, FII, and FIII (Figure-1). The protein content of the fractions FI, FII, and FIII was 54.6, 38.7, and 69.6 mg/ml, respectively.

### Electrophoretic profile of the isolated fractions

**Figure-2:**
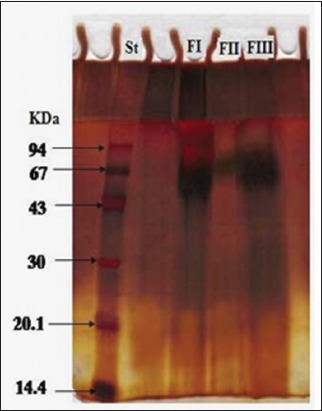
Electrophoretic profile of the three fractions of camel HCF antigen FI, FII, FIII, and Low Molecular weight standards (lane St).

FI migrates in two bands; a non-complex band with a molecular weight of 120 kDa and a complex band with a molecular weight of 60 kDa under non-reducing conditions in 12% SDS-PAGE. Conversely, FII revealed a complex band with molecular weight of 72 kDa and FIII revealed a complex band molecular weight of 74 kDa (Figure-2).

### Diagnostic potency of the three purified fractions

**Figure-3:**
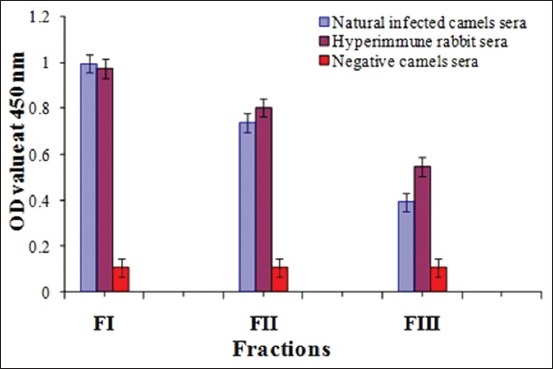
Comparative diagnostic potency of FI, FII and FIII.

Serum samples from naturally infected camels with *E. granulosus* and hyperimmune rabbit sera showed a strong reaction against these three fractions. Higher potency in the detection of antibodies against CE was observed in purified FI than in FII and FIII, as shown in Figure-3.

### Sensitivity, specificity, positive predictive value (PPV), and negative predictive value (NPV) of FI using indirect ELISA

Using ELISA, sensitivity of purified FI antigen against positive naturally infected camel serum samples was found to be 88%, and specificity was 91.7%. FI had PPV of 95.6% and NPV of 78.6%.

### Diagnosis of camel CE

**Figure-4:**
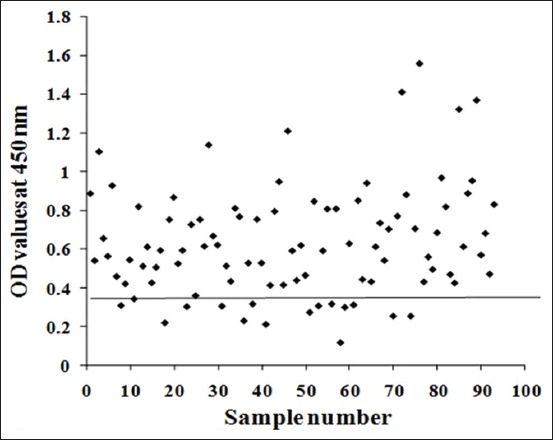
Diagnostic potency of FI in collected camel random serum samples.

Indirect ELISA was adopted to diagnose *E. granulosus* cysts in camel using purified FI as an antigen. FI antigen showed that 81.7% of randomly selected camel serum samples were infected with CE, as shown in Figure-4.

### Antigenic bands

**Figure-5:**
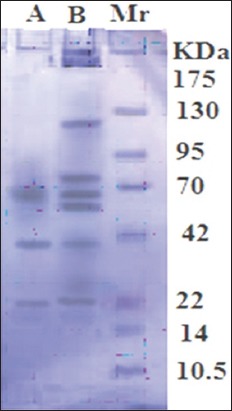
Antigenic bands under reducing condition recognized by naturally infected camel serum in crude HCF antigen (Lane B) and purified FI (Lane A). Standard’s molecular weight (Lane Mr).

The antigenic bands in crude HCF antigen were 120, 74, 60, 57, 38, 22, and 8 kDa (Figure-5 Lane B). The antigenic bands of FI antigen were identified as 60, 38, and 22 kDa using immunoblot under reducing condition, in which infected camel serum samples were utilized (Figure-5 Lane A).

## Discussion

In the present study, Sephacryl S-300 column chromatography was used to purify the HCF antigen collected from camel lung cysts. Three fractions, FI, FII, and FIII, were isolated. Indirect ELISA showed that FI was the most potent diagnostic antigen. This antigen contained two bands, a non-complex band with a high molecular weight of 120 kDa and a complex band with a molecular weight of 60 kDa, as confirmed using SDS-PAGE under non-reducing conditions. The previous studies recorded antigen 5 and antigen B as the most potent diagnostic antigens of camel *E. granulosus* cysts [30,31]. Antigen 5 has a high molecular weight of 400 kDa and dissociates into complex components of 60-70 kDa under reducing conditions [1,11]. This behavior is similar to that of FI, which showed a band with a high molecular weight of 120 kDa that dissociated into three antigenic bands at molecular weights of 60, 38, and 22 kDa under reducing conditions by immunoblotting with positive CE camel sera.

The appearance of these bands was possibly due to the dissociation of the high molecular band into a small band of 60 kDa and two smaller bands of 38 and 22 kDa under reducing condition. This result may be in agreement with that of a previous study by Monteiro *et al*. [32], which suggests that native antigen 5 complex has a large molecule of 600 kDa equivalent to an oligomeric structure of about 10 units. Correspondingly, antigen 5 is an oligomeric thermolabile glycoprotein that migrates as 57 and 67 kDa bands in SDS-PAGE under non-reducing conditions and as 38 and 22 kDa bands under reducing conditions [33]. Pagnozzi *et al*. [18] found that antigen 5 purified by fast protein liquid chromatography, analyzed by SDS-PAGE under non-reducing and reducing conditions, and examined by immunoblotting with positive CE patient sera yields reduced antigen 5 with 38 kDa band of light reactivity and non-reduced antigen 5 with 60 kDa band, which has the strongest reactivity. The 22 kDa antigenic band of antigen 5 results due to the presence of heparin sulfate proteoglycans binding sites [34,35]. These observations led to the comparison of FI with antigen 5 where both had high-molecular-weight complex dissociating into smaller entities of diagnostic potency of camel CE. In the current study, indirect ELISA was used for serodiagnosis of camel *E. granulosus* cysts recording 88% sensitivity and 91.7% specificity. These results matched those of a previous study by Pagnozzi *et al*. [19], which reported that antigen 5 ELISAs revealed different sensitivity (88.3%) without significant differences in specificity (92.5%).

The origin of FI from camel cyst is an additional advantage of FI. Based on molecular identification studies of animal and human isolates, camel cysts are more potent in causing infection in humans [36,37]. The Egyptian G6 strain nucleotide sequence was the predominant genotype in Egypt, and its involvement has been shown by several studies [38-40].

## Conclusion

The diagnostic antigen of camel origin can be successfully utilized for the diagnosis of *E. granulosus* cysts by ELISA, which may help control the infection and minimize transmission to humans. In addition, the antigen of camel origin could successfully be utilized in the diagnosis of human infection. A local HCF antigen FI isolated by an easy, one-step, low-cost purification method proved to exhibit high diagnostic potency for camel CE.

## Authors’ Contributions

All authors participated in the study design. NIT collected lung hydatid cysts and blood samples from slaughtered camels, prepared crude antigen, performed a hyperimmune rabbit serum and carried out Western blot. MSH performed a purification of HCF antigen by Sephacryl S-300 gel filtration column chromatography. MSH and EEE carried out SDS-PAGE under non-reducing conditions. NIT, EEE and EHA shared in ELISA for the detection of CE-specific antibodies. NIT and EHA analyzed the data. NIT and EHA participated in writing the manuscript. All authors revised and approved the final manuscript.
